# Book Reviews

**Published:** 1819-09

**Authors:** 


### CRITICAL ANALYSIS

OF

### RECENT PUBLICATIONS, IN THE DIFFERENT BRANCHES OF MEDICINE AND SURGERY.

"I would have men know, that, though I reprehend the easie passing over of the causes of things " by ascribing them to secret and hidden vertues and properties; (for this hath arrested and laid " asleepe all true enquiry and indications;) yet I doe not understand but that, in the practical " part of knowledge, much will be left to experience and probation, whereunto indication cannot " so fully reach: and this not only in *specie*, but in *individuo*. Yet it was well said, *Vere scire* " *esse per causas scire*."—BACON.

***Observations on the Symptoms and Specific Distinctions of Venereal Diseases; interspersed with Hints for the more effectual Prosecution of the present Enquiry into the Uses and Abuses of Mercury in their Treatment.*** By RICHARD CARMICHAEL, M.R.I.A. one of the Surgeons of the Richmond Hospital, House of Industry, &c. &c. 8vo. pp. 221, with a coloured plate. Longman and Co. 1818.

" He is a foolish physician who, instead of curing his patient's disease, doth cast him in another sickness."—MORE's *Utopia*.

THE diseases which form the subject of this work have many forcible claims on the most earnest attention of the medical philosopher. The history of their progress during the last three centuries displays, perhaps, the most afflicting series of calamities recorded in the annals of our art. Plagues and pestilence may have caused more extensive destruction of human existence; but, if we trace the physical effects of those maladies through all their direct and devious courses, and then add to these the moral ills consequent on those illustrations of the sad truth, that

——————e medio de fonte leporum
Surgit amari aliquid, quod in ipsis floribus angat; it will be evident that they have been one of the greatest scourges the genius of Evil ever sent forth to desolate mankind. But, we rather choose to turn to the prospect opened to our view, and contemplate the happy change about to ensue from the application of the true elements of medical logic to researches on the pathology and treatment of those diseases. We have an ample scene for this in the work of Mr. Carmichael.

The author commences with a concise but lucid view of the principal difficulties that have attended the investigation of the nature of these diseases, and the most serious of the errors into which pathologists have fallen in their mode of conducting their researches. We have, he says, been long taught to believe that mercury was the only remedy for every form of venereal disorder, gonorrhœa excepted. It was therefore time to commence the investigation, when every practitioner must have met with circumstances to shake his faith in the powers of the remedy, from perplexing embarrassments and inextricable difficulties accumulating upon him, as long as he persisted in the exhibition of this specific. But, he continues,

"In order to preserve our faith unshaken, ingenious devices were sagaciously resorted to. By one of these we learned, that not only the new symptoms which arose under the most severe courses of mercury, but even the old ones which resisted its influence, were attributable to the remedy and not to the disease. Hence we have descriptions of mercurial chancres, mercurial ulcers, pains, nodes, and swellings of the lymphatic glands of the neck. But, in ascribing those symptoms to mercury, we have entirely overlooked this obvious circumstance, that *that* medicine, when exhibited even to profusion for liver or any disease which is not venereal, has never in any one instance produced those effects."

Mr. Carmichael is willing to allow that, on the occasions just mentioned, mercury, although it has not the power totally to supersede the actions of the morbific poison, may yet so far alter or modify its effects, as to change the appearance and natural progress of the disease. But this, he says, "is different from an admission that the remedy will produce symptoms which can scarcely be distinguished from those of the poison itself."

This doctrine, although it is not satisfactory to the judgment, seems to be so far supported by observation as to lead us to consider it as a valid basis for our practical indications. It should be considered as a subject urgently requiring a new and particular investigation. But the most serious errors into which we had fallen, are now to be noticed.

"Another device, common to many arts and sciences besides surgery, is an endeavour to conceal our ignorance by the adoption of plausible and delusive epithets and appellations. The term syphiloidal I cannot but regard in this point of view: it is usually applied to those symptoms which continue to linger after the patient has undergone full and repeated courses of mercury, and which that medicine was found incapable of curing. Those, therefore, who looked upon mercury as a certain cure for every form of venereal disease, found it necessary to give those unaccommodating symptoms a name: they therefore called them syphiloidal, which, if it means anything, insinuates that something is present which resembles or appertains to syphilis, and which is not syphilitic. The coinage of this name, however, gave them an opportunity of relinquishing the further use of mercury, without making the mortifying acknowledgment that they had been using, to a dangerous extent, a medicine incapable of curing the disease for which it was exhibited. These subterfuges were however useful, and I will even say reflected some credit upon those who devised them, as they obviated the injurious perseverance in the use of the medicine, which might otherwise have been considered a matter of necessity.

"Since, however, it is now well known that certain forms of venereal disease will pursue their course whether mercury is employed or not, it is absurd any longer to retain words in our vocabulary, which are calculated to mislead us from the truth. Nothing, I am certain, would tend more to promote the present investigation, and the attainment of a perfect knowledge of venereal diseases, than to drop altogether those common but arbitrary terms, syphilitic, syphiloidal, and pseudo-syphilitic: even the term mercurial should be restricted to designate those phenomena only which are known to arise from the use of mercury in other diseases besides the venereal, by which means we avoid the perplexity of confounding the symptoms arising from the poison with the effects of the remedy.

"In place of those arbitrary names, which mean nothing, if surgeons would confine themselves merely to terms descriptive of appearances and symptoms, language would not be wanting to convey an adequate notion of any class of diseases."

Those sentiments are in conformity to the true principles of medical logic, and will be acceded to by every person who is able to discern the means by which medical knowledge can alone be accurately acquired, and displayed with clearness and precision.

The distinctions which Mr. Carmichael has observed in the appearance and progress of certain groups of symptoms, which usually went together in diseases apparently arising from venereal congress, led him to presume the existence of a pleurality of poisons, which are propagated in this manner. The best pathologists seem to be well inclined to concur in this opinion, which is not only supported by daily observation, but also by the most authentic historical records. It seems hardly to admit of a doubt that animal poisons have been casually called into existence, that have been diffused throughout whole nations by venereal congress, at various epochs; and we think the opinion of Dr. Weizmann, of Bucharest,* that these poisons have frequently originated on the connexion of different races of people, merits particular attention. Literary records will probably not furnish the means of fully deciding this question, but, as far as our researches have hitherto extended, they powerfully support the opinion under consideration. We cannot in this place indulge in details on this subject, but we may observe in general, that, at different epochs since the eleventh century, alarms have been spread throughout one or more nations of Europe, by the progress of some dreadful ulcerative disease, obviously propagated by venereal intercourse. For instances:—soon after the crusades, the dispersion of the Moors, the expulsion of the Jews from Spain, the discovery of America, the war in Canada. We may add, too, that many curious facts

* See *London Medical and Physical Journal*, vol. xli. p. 64.

respecting hybrid animals and vegetables, seem to lend some support to the same opinion.

Whether or not Mr. Carmichael has precisely determined the number of the species of poisons ordinarily propagated by venereal intercourse at the present time in this country, is a question we cannot pretend to enter into the discussion of; but this is not of much importance in the view in which he has regarded these diseases, which we cannot too much approve. His object is to determine the character, series, and order, of their symptoms; and the appropriate measures for their cure, especially when mercury should, and when it should not, be employed. This is a view of the subject which must be fully approved, even by those who consider that the varieties of character which Mr. Carmichael has distinguished, depend on peculiarity of constitution of the patient, not on a specific difference in the nature of the morbific poison.

Mr. Carmichael has, however, adduced evidence of the most powerful kind, although not sufficient to decide the question, to favour his distinctions of venereal diseases: he says,

"We observe many primary ulcers evince, from their very commencement, such peculiar and distinct characters, that it would be quite an absurdity to believe that the virus is always the same, and the variety of characters dependent alone upon constitution.

"Thus nothing can be more opposite, even from the commencement, than the common chancre with its hardened base, like a piece of cartilage under the skin, and the sloughing ulcer. The first is slow and chronic; the latter begins with a mortified spot, extends by alternate sloughing and phagedenic ulceration, and makes more progress in three days than the former in as many weeks.*

"The phagedenic ulcer is equally distinct from chancre, as it does not evince at any period a hardened base, but gradually creeps from one part to another of the penis, leaving those parts to heal which in the first instance it attacked. So that, when the disease has existed for some months, the glans is seen to exhibit its entire surface furrowed over with ulcerations and cicatrices.

"There is a raised ulcer also with elevated edges, approaching the nature of the phagedenic ulcer, yet whose characters are sufficiently distinct to be considered as a separate species.† But the most common venereal primary ulceration presents such various appearances in different individuals, that, until a more exact knowledge is obtained, it is better described by its negative than its positive qualities; and it may be designated an ulcer without induration, raised edges, or phagedenic surface.

* "A striking elucidation of this fact is afforded by Case lxviii. p. 162, of my *Essay on the Venereal Diseases which have been confounded with Syphilis*, &c.

† "For a more particular description and examples of this ulcer, see chap. iii. p. 62, of my former Essay; and also the *London Medical and Physical Journal*, for December 1815.

"If the plurality of venereal poisons is supported by the variety of primary ulcers, it is equally so by the multiplicity of constitutional eruptions. A primary ulcer, which was not phagedenic or sloughing at first, may afterwards, like any other ulcer, become so by irritation, neglect, or inflammation. But I do not conceive that we have grounds for supposing that the state of the constitution can so modify morbid poisons, as to cause the same virus to produce in one person the chronic scaly lepra and psoriasis, and to assume in another a decided pustular form, each pustule spreading rapidly into a deep ulcer.*

"These two kinds of eruption may serve to illustrate the subject, as in their nature they are so directly at variance. But I would be inclined to admit, that an eruption of papulæ with acuminated heads containing matter, and approaching the pustular form, might be so affected by the constitution as not to be distinguishable from the most regular pustules. The character of the disease may however still be apparent, as their pustules, instead of spreading into extensive ulcers, will, like papulæ, terminate in desquamation; but the scales will be larger; and, in addition to this circumstance, the pustules will throughout be intermingled with papulæ. These circumstances may serve to distinguish, in doubtful cases, the form of disease which is attended by the venereal lichen or papular eruption, from that which is much more formidable, and produces pustules which end in obstinate ulcers, that spread at their circumference by a phagedenic edge, and heal from their centre."

Pursuing the investigation of the progress of these diseases, Mr. Carmichael found that each of the species of primary ulcer above described was followed by a certain and peculiar order of secondary symptoms; and in the following manner:

"*First.*—That the syphilitic chancre is attended by the scaly eruptions lepra and psoriasis, an excavated ulcer of the tonsils, and pains and nodes of the bones.

"*Second.*—That the ulcer without induration, raised edges, or phagedenic surface, gonorrhœa virulenta, and excoration of the glans and prepuce, are followed by a papular eruption, which ends in desquamation, pains in the joints resembling those of rheumatism, soreness of the fauces, and frequently swelling of the lymphatic glands of the neck; but that, in a vast number, not a single instance was observed in which nodes were an attendant upon this eruption.

"*Third.*—That the ulcer with elevated edges, in the few instances in which I had an opportunity of tracing it to its constitutional symptoms, was followed by a pustular eruption, which terminated in mild ulcers, pains in the joints, and ulcers in the throat, but no appearance of nodes; yet that the instances in which I had an opportunity of witnessing distinctly the connexion between the primary and secondary

* "The reader may compare the different species of eruptions delineated in the plates of my former Essays; but particularly let him compare the scaly chronic syphilitic eruption in plate i. with the acute pustular eruption in plate iv. These forms of eruption are as dissimilar in their progress as they are in their appearance.

symptoms of this poison, were too few to form any conclusion with respect to this particular.

"*Fourth, and lastly.*—That the phagedenic and sloughing ulcers are generally attended by constitutional symptoms of peculiar obstinacy and malignancy; viz. pustular spots and tubercles, which formed ulcers that spread in general with a phagedenic edge, and heal from the centre. Extensive ulceration of the fauces, particularly of the back of the pharynx; obstinate pains of the knees and other joints; while nodes are frequently present, and the bones of the nose are occasionally affected."

After passing in review the opinions of Mr. Rose, Mr. Guthrie, Dr. Thomson, and Mr. Hennen, respecting the cure of the syphilitic species of disease without mercury, the author relates the results of his own experience. We may now pass over this part of the work, since it engaged our attention on a former occasion, (see vol. xli. p. 60 *et seq.*) to the chapter on *Iritis*.

In his former essay on Venereal Diseases, Mr. Carmichael expressed himself disposed to consider *venereal iritis* as a consequence of the poison of *syphilis*, or that which occasions the *chancre* (of John Hunter) and the *scaly eruption*, because he had always seen it yield quickly to mercury, and he had not then seen this affection accompanied with any eruption. Since that period he has met with many cases of this combination of symptoms, and, with one exception, the eruption was always papular: of this, several cases are adduced. These observations are particularly interesting; they serve to elucidate an obscurity in the history of iritis, which so much embarrassed Mr. Travers in his researches into the nature and cure of this affection. We cannot follow the author through all his judicious and highly valuable reflections on this subject; but, by way of summary, it may be stated, that it appears, from Mr. Carmichael's observations, that iritis is a symptom of the disease attended with the papular eruption, which disease will run its course in spite of the use of mercury, although some of its symptoms may be removed by that remedy; that it will occur with the papular eruption, when no mercury has been used; that it will happen without the presence of any eruption; and that, in either case, the use of mercury, although not essentially necessary for its cure, will be beneficial in conjunction with antiphlogistic measures, by assisting in removing the inflammatory action, and by preventing its disorganizing effects.

The subject of the next chapter, the *phagedenic ulcer*, is the most serious, and hitherto the least understood, of the forms of venereal disease. We may, in the first instance, refer the reader to the thirty-fourth volume of this Journal, for Mr. Carmichael's description of the character of this ulcer.

The origin of this from a peculiar species of morbific poison, has been disputed by some pathologists. They consider its phagedenic character to depend on the state of the constitution of the patient; and state, they have observed many cases of venereal ulcer, of the ordinary character on their first appearance, which have subsequently assumed all the characters of the ulcer designated by Mr. Carmichael as the primary phagedenic ulcer, and which have also been followed by the eruption he considers peculiar to that species of primary affection. This objection has been expressly made by Mr. GUTHRIE. This is an important objection, because, what forms the most powerful evidence of the propriety of Mr. Carmichael's distinctions, is the constant and regular connexion of the primary and secondary symptoms which he has described. In reply to this, the author states, that in every instance where he has had an opportunity of tracing the constitutional symptoms to the primary ulcer, this ulcer has always exhibited the phagedenic character; and it should be observed, that Mr. Guthrie acknowledges that the ulcers he refers to became afterwards phagedenic.

There is much difficulty attendant on the investigation of this question, which, as Mr. Carmichael readily acknowledges, requires further observations to enable us to come to a decision. These ulcers, he remarks, are not often seen in their first stage, generally not till some days, or even weeks, have elapsed, in consequence of the little uneasiness they then occasion; and to this must be added the fact, that various accidental causes will make any species of ulcer become phagedenic. The constitutional symptoms that Mr. Carmichael has seen ensue from ulcers of the latter kind, have however been of a mild character, and readily admit of cure; whilst those that have occurred after the primary phagedenic ulcer are always of a formidable character, and long and difficult of cure.

We, on a former occasion, mentioned that Mr. Carmichael had some doubts respecting the power of mercury to prevent constitutional affection from the syphilitic primary ulcer; he is still less disposed to place confidence in the efficacy of that medicine to prevent the constitutional symptoms of the phagedenic disease. He does not absolutely protest against its use; but, if it be employed, it should not be resorted to until the primary ulcer is perfectly healed. It is decidedly injurious in all cases in the primary affection. This latter point is generally agreed on by experienced surgeons; and wine, bark, and opium, are the remedies on which they have usually placed their reliance, until its phagedenic character has disappeared, and the ulcer, if not quite, at least very nearly, healed. Mr. Carmichael places most reliance on the efficacy of copious blood-letting in the treatment of this disease. He has frequently seen cases where the ulcers spread rapidly under the use of the remedies we have mentioned, but ceased their ravages on the occurrence of a spontaneous or artificial hæmorrhage. With this he has usually conjoined antimonials, purgatives, cicuta, and opium. Warm fomentations and bread-poultices, frequently with the addition of opium, are recommended, as local applications. In every instance, low diet and the recumbent position should be strictly enjoined.

We now enter on the consideration of the secondary symptoms, which the author considers to be peculiar to the species of primary ulcer just described.

The eruption is frequently ushered in by a high degree of fever, and it "quickly passes into ulcers covered with thick crusts, that heal from the centre, whilst they are extending at the circumference with a phagedenic border." To this description we shall add some particular observations that our own experience has afforded, which we think may be useful to the young practitioner, in assisting him to form the diagnosis of this affection. Whether it is the result of the influence of a distinct poison, or of syphilitic virus modified by particularity of constitution, may admit of doubt; but it is certain that there is an affection, offering constantly the morbid characters here detailed, which is always formidable in its progress, and for which mercury is not an efficacious remedy. Reasoning by analogy on these data, would lead us to consider it as a distinct disease.

The eruptions, a few days after their first appearance, we have usually found to be about the size of a pepper-corn, not quite so prominent as the pustules of small-pox, forming about half a sphere; they are not surrounded by much diffused redness in the first instance, but this appears generally at a later period; the interstices are then very red, or of a purplish colour. The arms, neck, face, and shoulders, are the parts chiefly, and almost simultaneously, affected. The ulcers are very painful, and sometimes spread to a diameter of one or two inches; often covered with fungus; and, when clean, have a scooped appearance. We have a suspicion that this disease may be communicated by these secondary ulcers, from having witnessed an ulcer on the prolabium of a female, obviously produced from contact with the matter of the secondary ulcers just described, which assumed all the characters of the primary phagedenic ulcer, and caused a considerable destruction of parts, before it was induced to heal by the exhibition of wine, bark, and opium. It should be observed, that there was a slight breach of surface on the lip before the contact of the morbid matter. We could not ascertain whether or not this patient had secondary symptoms, having lost sight of her soon after the healing of the ulcer.

The affection of the throat, Mr. Carmichael observes, that attends the phagedenic disease, is of the most formidable character: it attacks more particularly the back part of the pharynx, sometimes passes upwards to the nares, and occasionally extends to the epiglottis and the larynx. The ulcer is mostly covered with a thick, white, tenacious, matter, and is attended with considerable difficulty in deglutition. The ulceration often extends to the adjacent bones. It is this disease which causes the destruction of the bones and cartilages of the nose. Mr. John Pearson used to state, in his lectures on the venereal disease, that he had seen only two or three cases where the nose had been destroyed by syphilis, when suffered to proceed uninterrupted by art; but a great many by the influence of mercury. Mr. Carmichael has shown us how this circumstance should be regarded; that it is not from the influence of mercury alone that this destructive ulceration has ensued, but from mercury having been employed in the treatment of the phagedenic disease, and generally to a very great extent, in the hope of thus deriving benefit from it, after the ordinary measures had been used in vain: finding, then, the disease exasperated by the use of the medicine, the surgeon was led to consider it as the consequence of its agency.

Inflammation of the parts constituting the joints, especially of the synovial membranes, is another frequent affection in this disease.

"Nodes were only traced to the phagedenic primary ulcer in such cases as were treated with mercury. Both Mr. Guthrie and Mr. Rose have stated the remarkable infrequency of this symptom in numerous cases which were treated without that medicine. The question, therefore, whether they occur in this form of disease where mercury has not been exhibited, remains to be resolved by future experience.

"The train of symptoms I have been endeavouring to describe, is so often found to be injured rather than benefitted by the exhibition of mercury, that we may well ascribe to this circumstance the coinage of the favourite terms mercurial, (in the unrestricted sense in which it is employed,) syphiloidal, sequelæ, &c. &c. and I have no doubt but that this form of venereal disease has at length led to the present investigation, to ascertain how far venereal complaints can be combated without the exhibition of mercury; an investigation, which, in spite of the common-place declamation and untractable dogmatism of professional bigots, will, I am persuaded, lead to an improved line of practice, and confer the most important benefits upon society."

Mr. Carmichael repeats, that he has not a doubt but that the embarrassing obstinacy of this disease arises from the premature and indiscreet interposal of mercury, which interrupts and encumbers its natural progress, and transfers it to the deep-seated parts,—as the periosteum, fasciæ, and bones.

The synopsis of the pathology and treatment of venereal diseases according to the doctrines of Mr. Carmichael, (which is transcribed in another part of this Journal,) will furnish a general account of his precepts for the treatment of the constitutional symptoms of the phagedenic malady. We shall here only observe, that mercury is of but occasional and partial utility; is more frequently deleterious than beneficial; although it sometimes alleviates some of the symptoms, it will not check the progress of the disease; and new symptoms commonly appear, when the system has been long under the influence of that medicine.

This disease in many respects resembles yaws; and the author refers to the work of Dr. Adams on Morbid Poisons, for some interesting facts and valuable observations illustrative of this resemblance.

We cannot follow the author through all his reflections on the mode of treating the different forms of this disease; they can only be contemplated with all the benefit they are calculated to produce, in connexion with the cases which accompany them, which serve as illustrations. We must also remark, that the value of the doctrine contained in this work is much increased by a view of it which now forcibly presents itself. The author has methodically illustrated his principal points of theory by appropriate cases, which render his descriptions perspicuous, and at the same time show the bases on which they are raised.

The diseases of a less-distinctly marked character, though resembling in some respects the phagedenic malady, is next considered. On this subject we do not find many observations of important novelty, after the history Mr. Abernethy has given of those affections.

This work concludes with some critical remarks on the opinions of Mr. Charles Bell respecting the nature and treatment of venereal diseases.

From the account we have given of this treatise, it must, we think, be evident that it is calculated to be productive of much benefit to medical science. Although the pathological distinctions the author has formed of the diseases that form the subject of it should not prove to be precisely correct, a question which remains to be decided by further experience, his accurate and perspicuous histories of those affections will not be the less valuable, either in a nosographical or therapeutical point of view: all other objects are of far inferior importance. The true principles of medical logic which have guided the author's researches, and the methodic manner in which he has displayed the results of his labours, may serve as a model to those who may continue the investigation of the history of the same maladies.

*Practical Observations on the Causes and Cure of Insanity.* By WILLIAM SAUNDERS HALLARAN, M.D. Physician to the Lunatic Asylum of Cork, and a Corresponding Member of the Association of the King's and Queen's College of Physicians, Dublin. Second edition. Edwards and Savage, Cork; and Longman and Co. London. 8vo. pp. 213. 1818.

"Nisi sanatus animus sit, quod sine philosophia fieri non potest, finem miseriarum nullam fore."—CICERO.

"THE cloud of darkness and uncertainty which still, unfortunately," says the author, "pervades this department of medical science, is deeply to be lamented. An extraordinary neglect on the part of physicians of due attention to the subject of insanity, bespeaks a supineness for which there cannot be any admissible excuse."

We acknowledge the truth of the sentiment expressed in the first of the foregoing sentences, and shall have occasion, before we have concluded our review of this work, to notice some of the most powerful of the obstacles which have prevented the elucidation of the nature of this malady. But, that insanity has been neglected by physicians of modern ages, is not so evident. They have in general devoted as much attention to this subject as could be thus beneficially applied. The nature of the affection, from which the patients of it are ordinarily collected into particular receptacles under the superintendance of a few individuals, must however deprive the generality of physicians of the means for making original observations; but, that those who have possessed this advantage have not failed to employ it in a proper manner, is so clearly shown by the medical literature of Europe, as to render a formal demonstration of it unnecessary.

We pass over the rest of the introduction, since it contains nothing worthy of remark, except some erroneous doctrine respecting "mental insanity," that we shall not meet, with more fully displayed, in the author's "*pathological division of insanity.*" Dr. Hallaran commences this subject with stating that

"A principal object of this treatise is, to point out the practical distinction between that species of insanity which can evidently, *ab initio*, be referred to mental causes, and may therefore be denominated mental insanity, and that particular excitement, which, though partaking of like effects, so far as the sensorium is engaged, might yet appear to owe its origin merely to organic disease. Authors on this subject seem, heretofore, to have passed over this necessary consideration."

The principal object of this treatise is, then, to advance a point of doctrine, which has not been excelled in absurdity by any of the chimerical dogmas that error has in any age introduced into medical reasoning. The invention of such a doctrine a few centuries since, when almost every branch of science was cultivated on erroneous principles, cannot excite our surprise; but it does this, to find it brought forward at a period when the influence of the works of BACON, of LOCKE, and of CONDILLAC, is generally diffused amongst men engaged in the pursuit of science. Bacon teaches us, that the only knowledge we can acquire of nature, is the history of phenomena evident to our senses; and the metaphysical principles of Locke and Condillac, principles acquired in the mode inculcated by Bacon, declare the notions advanced by Dr. Hallaran respecting the human mind to be utterly erroneous. This is the conclusion our best principles of metaphysics lead us to form respecting the proposition, that intellectual insanity can exist independant on corporeal disorder. But let us examine the subject in a physiological point of view; or rather, the arguments of this nature produced by Dr. Hallaran to support what he pleases to consider as his own original doctrine. We shall not enter into a general discussion of it, because it is of a very ancient date, and is now, we believe, almost universally abandoned. As far as we can learn from literary records, it was first adduced by PLATO, (see his *Timæus* and *Theatetus;*) but the Grecian physicians, cultivating physiology on the principles of HIPPOCRATES, would not admit it: it was again brought forward when the philosophy of DES CARTES, or rather of PEREYRA, came into vogue; and was finally extensively displayed by Dr. ARNOLD, in his treatise on Insanity. These observations will serve as our apology for not entering into a regular discussion of this subject.

The author, as his first argument, says,

"I would advert to the many well-authenticated instances of insanity, as they have occurred within the last twenty-five years especially, and are noted on the records of our lunatic asylum. Amongst those are several which have owed their origin to mental causes, strictly speaking,—such as dread of punishment, loss of friends, shame, sudden terror, remorse, &c."

Every physiologist knows (it is proved by dissection) that dread, terror, remorse, and all the passions, are accompanied with inordinate cerebral actions; and LOCKE teaches us, that these inordinate actions only arise, either immediately from the impressions of external objects on the organs of sense, (to which modern physiologists are disposed to add those from the viscera, &c. but this does not alter the question in dispute;) or, subsequently, from the same actions being reproduced by the agency of memory. Dread, grief, shame, terror, remorse, &c. and their consequences, are then essentially dependant on corporeal disorder. Permanent or remittent insanity is sometimes the result, because those causes may induce either functional or organic lesion.

The author again says,

"The influence of terror on the human mind suddenly induces uncontrollable madness, without seeming to disturb any of the principal organs of vitality."

We have never seen a case of this kind, and we do not think there is another physician in existence who believes that it can possibly happen. If Dr. Hallaran will say that he has seen instances of it, we will say that he has seen inaccurately. But let us proceed: Dr. Hallaran says,

"I cannot question the immateriality of the mind or soul; but I will not deny that the mind or soul sickens. So long as the soul remains incorporated with man, so long will it partake of his nature, according to his attributes."

And again,

"The diseased manifestations of the mind, arising from terror, grief, excess of joy, remorse, shame, loss of property, and despair, prove the sensibility of the mind, though they betray its weakness. To deny the influence of those contending passions, would be, in some respects, to stifle principle, and to sink man below the level upon which Omnipotence had thought fit to place him. If the mind never sickens, conscience, the great inquisitor and monitor of man, would prove of no avail. Yet, this conscience, this mind, this soul, of man, is but too often most sorely afflicted. It encounters disease with a mighty struggle, and is sometimes triumphant. If the wound be deep, however, it will 'fall sick;' and with this sickness will come death. 'The spirit of a man will sustain his infirmity; but a wounded spirit who can bear?' (*Proverbs*, ch. xviii. v. 14.)"

This is such utter and irrelevant nonsense, that we almost criminate ourselves for having thought the author's arguments on this subject worthy of our notice. But, as we have commenced, we will proceed:

"The mind is capable of impressions which evidently disturb its natural functions: it will continue thus disturbed for a series of years, and not impart any ostensible disease to the organs of life; it will be partially or generally affected, and will frequently, without any strong effort, resume its original character."

Those impressions (corporeal, of course; or, if not, what can they possibly be?) which disturb the natural functions of the mind, are disordered, or diseased, impressions. No action that destroys the harmony of the economy can be considered as an healthy action. That insanity exists for a series of years, without producing "any ostensible disease in the organs of life," is very doubtful. But this is not a point of material importance, because it is well ascertained that functional derangement, esspecially deranged circulation, will exist in the brain for years, without dissection furnishing ostensible disease. We can point out sufficient proof of this fact, and at the same time direct the attention of the reader to an excellent practical work, by referring to Dr. PARRY's *Elements of Pathology.*

We will not argue on the absurdity of supposing that an immaterial existence can be *partially* diseased, and have efforts in itself by which it diseases itself, and then by an effort restores itself to health. We wish to consider the subject physiologically.

Dr. Hallaran proceeds to say,

"If the mind, under those circumstances, be not diseased, how are we to account for the relief which has been so repeatedly obtained, through agencies that cannot be supposed to influence any other than the perverted powers of the intellectual faculties?"

The influences to which the author here alludes are moral influences, which act on the mind through the medium of the brain, and may casually relieve the disease of the brain causing diseased manifestation of the intellects, just as the same influences will remove the disease of the brain, causing *epilepsy*, and even lesions in the functions of more remote parts, appearing as chorea, hysteria, ague, &c.; instances of which occur almost daily to the observance of the physician. A remarkable instance of this kind is that which BOERHAAVE relates of his curing, simultaneously, a whole troop of young girls, in an orphan asylum, affected with epilepsy, by threatening to burn them if the fit again occurred, and exposing the red-hot iron to their view. The great excitement thus produced in one portion of the brain, may probably remove that existing in another, as cauteries, &c. do diseases of parts adjacent to those to which they are applied, or those distantly situated, by sympathetic or nervous influence.

Dr. Hallaran, pursuing his course, adduces the following case in support of his hypothesis:

"Mental delusions, inciting to suicide, have often occurred, and, to my knowledge, are now in danger of occurring, where no bodily infirmity has been ever ascertained to account for such a propensity. The present justly-celebrated Dr. Gregory, of Edinburgh, was accustomed to relate a case, in his clinical lectures, very much in point, of a man, who, in a fit of insanity, had determined on self-destruction; and who had escaped from his house in London, at night, with the determination of precipitating himself from Westminster-bridge into the Thames. When about to complete his purpose, he was suddenly assaulted by an armed footpad, who threatened him with instant death. This not being the mode by which he had purposed to part with life, alarm for his safety instantly seized him, to the exclusion of the hallucination which had been but the moment before predominant. Being freed from his unsought danger, he, with altered sentiments, returned to his family, fully impressed with the criminality of his design, as well as relieved from the pressure of his previous perplexity.

"Are we, from this well-authenticated fact, to infer that the individual alluded to had been, at the time of so sudden a transition from disease to health, a sufferer from the consequences of mere corporeal ailment? What form of bodily complaint could have been urgent, which, with such unexampled rapidity, was disposed to give way? We do not in general find diseases, arising from organic lesion, disposed to assume the healthy aspect with this convenient speed. The mind, we must here admit, had sickened, and was diseased."

The remark we made respecting the cure of epilepsy, will also explain the nature of this case; and though "diseases arising from organic lesion *are not* disposed to assume the healthy aspect with this convenient speed," diseases arising from functional derangement are thus commonly relieved; especially in the brain, where a little inordinate vascular action will produce great disturbance of its faculties. As we like to support our opinions by those of others, we refer for proofs of this statement to a work in the possession, we hope, of every medical student,—the *Elements of Pathology*, of Dr. Parry.*

"The sequel of this case," (of the man who intended to drown himself,) says Dr. Hallaran, "enforces the conclusion, no proof of corporeal or organic injury had been alleged." We allege again, that the existence of insanity was a proof of organic injury; and the want of evidence of this injury by other symptoms proves nothing, because it is so common to meet with organic lesion in the brain when it has not been suspected by every physician.

We have noticed all that the author has produced, bearing the semblance of argument, in favour of his hypothesis of the "mental origin" of insanity: we shall now extract a passage from his work, in which he himself refutes his own propositions.

"Doctor Spurzheim admits, that 'certainly the manifestations of the mind may be deranged.' Those manifestations I am disposed to consider as the essence of the mind at the time being. If the manifestations of the mind be diseased from any given cause, they naturally must betray an unsound mind, in the ordinary acceptation."

Now, if the manifestations of the mind be the essence of the mind, ("at the time being" is a vague and unmeaning expression, because the essential quality of a thing is its quality at all times and on all occasions,)—the essence of the mind must be

* We must here remark, that we speak with such approbation of this work, because of the multitude of accurate observations it contains relative to the phenomena of disease: these are equally valuable, whatever opinion may be held of the physiological basis on which the doctrine contained in that work is raised.

corporeal actions, for every physiologist must acknowledge that such actions essentially constitute the manifestations of the mind; then, as a postulate, a deranged mind consists essentially in deranged corporeal actions; and the profound deduction! that diseased actions betray diseased actions, no one will object to. This is the author's doctrine, not our's: we are not such confirmed materialists.

We but rarely find persons give so ill an exposition of their own doctrines as Dr. Hallaran has done of his on the nature of insanity. We in general see them, after *eight years*' reflection, able to attribute a plausible character to principles almost as chimerical as those forming the basis of this work. Bijoux, a keeper in the menagerie at the Jardin des Plantes, who formed a classification of animals from the shape and colour of their excrements, could make a very profound and logical oration on the merits of his system, and it was quite impressive to hear the ingenious arguments he would produce to show its propriety; but, what is very extraordinary, and indeed unprecedented in the annals of science, he died (poor fellow!) without leaving behind him an apostle, to teach and perpetuate the knowledge of his discoveries.

Although we were to join Dr. Hallaran in his lamentations over neglected psychology, we could not recommend the results of his labours, to vindicate its cause, to the attention of our readers: in the want of all the existing dissertations by ancient and modern physicians, we would rather study the *Alma* of Prior; there is in that at least as good logic, and certainly much better physiology, than in the treatise of Dr. Hallaran.

The work of Dr. Hallaran, then, we observe with regret, tends much to favour the progress of pure empiricism: it shows that, without correct physiological principles, a person may in some cases be a good medical practitioner; for, the lunatic asylum over which he presides, is remarkable for the great proportion of cures, (especially when the causes of the disease in a great number of the patients therein admitted are considered,) effected within its walls; and we know that the moral and medical management, is there conducted with great propriety, so, indeed, as to be strikingly evident, in the former respect, to the patients themselves when convalescent.

If we were to indulge in a particular account of that part of the work which relates to the treatment of the disease, we should have much to notice with terms of warm approbation; we here find the remarks and precepts of a good practical observer, and feel increased regret that the assumption of erroneous psychological and physiological principles, should have led the author into the erroneous reasoning contained in the part of the work that has passed our particular review.

We must however describe the arrangement of the subjects, which is good in a practical point of view. Dr. Hallaran considers, in order, the general and local causes of insanity; its hereditary nature; the prognosis in its various forms; the method of cure in general; and then the particular remedies, as venesection, local blood-letting, emetics, purgatives, the circular swing, digitalis, opium, camphor, restraint, vesicatories, mercury, warm and cold bathing; the treatment during convalescence, and in the chronic form of the disease; the efficacy of spirit of turpentine in maniacal epilepsy; and he concludes with some reflections on the moral management.

Those subjects are treated with much precision, and with a spirit which shows the man of good talents, especially of acute observance. The knowledge of the results of the author's experience will be of great utility to those who are particularly interested in the acquisition of information respecting the treatment of this disease. For the illustrations of these remarks we refer to the work; an abstract would not place them in a proper point of view.

---

*The Dublin Hospital Reports, and Communications in Medicine and Surgery.* Vol. II. 8vo. pp. 396. Hodges and M'Arthur, Dublin; and Longman and Co. London. 1818.

[*Continued from p.* 144.]

*On a Disease of the Lymphatic Glands of the Groin, attended with peculiar Symptoms.* By A. Colles, M.D. one of the Professors of Anatomy and Surgery in the Royal College of Surgeons in Ireland, and one of the Surgeons to Dr. Steevens's Hospital.

This disease usually occurred in men between the ages of twenty and forty; Dr. Colles only saw one instance of it in a female. It appeared in the form of a chronic bubo, arising without apparent cause, suppurating very slowly, and remaining open for several months, attended with but very little pain. The remarkable symptoms were the constitutional affections.

"From the very earliest period at which I have had an opportunity of observing this complaint, the constitution is found to be engaged. The patient is affected with head-ach, which is more severe in the morning, and which is increased by stooping: he also admits, when questioned, that he feels more fatigue than usual from long-continued or violent exertions; his pulse is quick, being in no case, when he is out of bed, under 100, and generally beating 120 in the minute. This quickness of pulse appears the more extraordinary, as it is obviously not produced by a high degree of pain, nor is it accompanied by a discoverable derangement of any other of the functions: on the contrary, the countenance is natural, respiration easy, skin of temperate heat and not very dry, tongue clean, appetite as good as usual, and scarcely ever nocturnal sweats. The patient, however, feels himself more comfortable in the open air than when confined to the house.

"In the treatment, I have confined myself to those means which I have conceived to be calculated to mitigate the severity of the symptoms, and to promote suppuration, which indeed seemed to be generally an unavoidable, and always a salutary, termination of the disease. The head-ach appeared to be alleviated by no class of medicines but by purgatives. These were repeated every day, or every other day, until this symptom was completely removed: very large doses were often required to produce the desired effect. The removal of the head-ach was not attended with a diminished frequency of the pulse. Poultices, warm fomentations, and gum-plaisters, were the only topical applications to which I had recourse. Leeches had been applied, in two instances, before I saw the patients, but apparently without any salutary effect. Cold and (as they are termed) repellent applications, when used for a few days in the earlier stages of the disease, did not appear to produce either benefit or injury."

*An Account of an uncommon Disease of the Hand and Fingers.* By C. H. Todd, Member of the Royal College of Surgeons in Ireland, &c.

This paper contains some observations of remarkable interest; and Mr. Todd has viewed the subject in so accurate and judicious a manner, that we shall transcribe his account of the nature, and his opinions respecting the origin, of the disease to which they relate.

"As far as I have observed, this species of whitlow occurs only in persons who have passed the meridian of life, and in such as are weak and unhealthy. It is preceded by symptoms strongly indicative of great debility, and of a want of energy in the functions of assimilation. Patients complain for several days of loss of appetite, of flatulence, thirst, and irregularity of the bowels, depression of spirits, and watchfulness; then the local affection takes place, and is most commonly felt at night for the first time, the patient's restlessness being increased by stinging pains in the fingers or hand. At this period small red or livid spots, without hardness or elevation, may be observed, which soon become black. The sensation in the part is between soreness and itching, and the patient is induced to rub or scratch it: this accelerates vesication, the cuticle becomes detached, and a thin and offensive sanies is effused under it. When the vesicles are removed, the subjacent skin appears sphacelated, and superficial ulcers are discovered; the disease showing a disposition to extend by destroying the surface merely. At first, local pain is severe; but in a few days it is not much complained of, and the absence of pain is to be considered rather as a cause of alarm than the contrary. Several parts of the fingers and hands are liable to be attacked by this disease in succession; and it often happens that, when one part is nearly well, another will become affected. This must be expected in any case in which general indisposition continues.

"During the entire progress of this disease the patient labours under a low fever; sometimes this fever does not require confinement to bed, but in many cases it is serious, and is liable to assume a typhoid character. In every instance the functions of the stomach and bowels are imperfectly performed, and all the secretions are diminished: lassitude, mental depression, and anxiety, are among the symptoms most distressing to the patient and to the persons around him.

"The treatment necessary for a disease such as the paronychia gangrenosa appears to be, is so obvious, as to render a minute detail of it at present quite superfluous. However, I may observe, that in this affection our practice must be chiefly directed to counteract constitutional disease and weakness, and to improve the condition of the organs of digestion. Unless these objects are attained, our patient will, in all probability, sink, not under the effects of local irritation, but in consequence of that highly morbid state of the system, of which the topical affection is solely an indication. Before the disease appeared to me in this light, I met with two or three fatal cases of it, in which the affection of the fingers was not of an extent sufficient to account for the unfortunate termination of the complaint. Indeed, I never saw an instance in which the severity of constitutional indisposition could be explained by the local symptoms."

The author relates several cases, which seem to show the truth of his opinions; which are also, in no small degree, supported by our knowledge of the functions and connexions of the ganglionic system of nerves.

*Account of a Diseased Appearance in the Intestines of Children.* By JOHN CRAMPTON, M.D. Professor of Materia Medica, one of the Physicians to Steevens's Hospital and to the House of Industry, &c. &c.

These observations were made on the bodies of children who died in one of the fever-hospitals of the House of Industry. We transcribe the following passage, as containing some remarks of great practical interest:

"That the epidemic has throughout shown a strong tendency to attack the digestive organs, chiefly in their mucous coats, is fully admitted by most persons who see those fevers on a large scale: it has been sufficiently insisted on by Dr. Cheyne in his report, and an attention to this point tends to improve the practice in fever, where the epigastric distress is present. It shows that we should not confine our views merely to the sensorium, as some have done, but that we should extend them to other organs. Of the distress in these latter, the symptoms will often afford sufficient evidence, but the examination of those who die of such fevers will frequently put the matter beyond a doubt: indeed, the diseased appearances vary so much in those who die from fever, that it is natural to conclude that no particular mode of treatment can be equally suited to fevers, as they occur attended by topical affections in different organs. The lancet and smart purgatives, so useful in certain forms of incipient fever, are by no means equally suited to such as are attended with gastric and intestinal distress, except in those instances where the serous membrane of the abdomen becomes inflamed,—an occurrence which may take place at any period; and then it will be necessary to have recourse to general as well as local sanguineous depletion."

It should however be observed, that Dr. John Crampton thinks it by no means clear, that the appearance forming the express subject of these observations, should, in every instance, be considered as the cause of the form of fever which happened to be present: it accompanied the epidemic of the season, it likewise attended the small-pox, and it was observed, in some instances, independent of any febrile disorder. The appearance, he remarks, was similar to that described by Dr. BAILLIE, under the head of dysentery;* a description of it will be found also in MORGAGNI,† and in the Transactions of the Berlin Medical Academy.‡

This paper contains many judicious remarks on the importance of attending to a subject much neglected in this country,—the state of the mucous membranes in febrile disorders; and, had not this been so extensively treated in the *Exposition of the Doctrine of* M. BROUSSAIS, we should have been disposed to adduce several extracts from the valuable memoir of Dr. John Crampton.

*The History of a Case of Gun-shot Wound of the Head; in which a Portion of a Bullet, &c. lay in the Substance of the Brain for several Months, without the Mental or Physical Powers of the Patient being injured.* By JOHN KIRBY, A.B. Member of the Royal College of Surgeons in Ireland, &c. &c.

This was an instance of a pistol-bullet entering the cavity of the cranium. When the immediate consequences of the injury subsided, the patient continued tolerably well for about six months; but symptoms of irritation of the brain then appeared, and he died about seven weeks afterwards. The following is the account of the appearances on dissection:

"Directly opposite the sinus in the scalp, there was a perforation in the os frontis, sufficiently large to admit the point of the little finger, and a groove in the external surface of the bone leading towards the wound, which had been made for the extraction of the ball.

"The internal spine seemed as if it had been broken, pushed aside, and had again united; for the bone was extremely rough, spiculated, and prominent in this part.

---

* *Morbid Anatomy*, p. 179; *Baillie's Engravings*, fasc. iv. plate 3, fig. i. and ii.

† *Lett.* xxxi. art. 21.

‡ *Acta Med. Berolin.* Dec. i. vol. ix. p. 69.

"The dura mater, through which there was an opening corresponding with that in the bone, was morbidly adherent to its margin. The pia mater was also pierced, and closely united to the dura mater, in the vicinity of the sinus, which led to an abscess in the left hemisphere of the cerebrum, containing something more than an ounce of pus, and a large ragged portion of a bullet.

"There were several pieces of bone within the substance of the brain at different distances from its surface, and some had passed altogether through it, and lay below the hemisphere. The ventricles contained upwards of a pint of fluid. The remainder of the brain was remarkably firm, and free from all appearance of inflammation."

*A Case of Disease of the Gums, which occurred during Pregnancy.* By J. PITCAIRN, M.D. Member of the Royal College of Surgeons, London, and Deputy-Inspector of Military Hospitals.

We discern nothing sufficiently interesting in this case to merit any particular remark.

*Two Cases of Ruptured Bladder from Accident.* By J. W. CUSACK, M.D. one of the Surgeons to Dr. Steevens's Hospital, and Surgeon to St. Patrick's Lunatic Asylum.

The patient of the first case was a man, aged 26 years, who fell in walking whilst the bladder was distended with urine. The usual symptoms of the effects of extravasated urine ensued, and he died eight days after the accident. An opening was made in the abdomen on the third day, to give vent to the diffused fluid. The appearances on dissection were signs of severe and general inflammation of the peritoneal membrane. The bladder was found ruptured on the right side, a little posteriorly, to the extent of an inch in the contracted state of the organ.

In the second case, a man aged 30, it is remarkable that the bladder had been evacuated a short time before the accident: the patient fell from the battlements of a bridge, about twenty feet high, to the ground, and he himself stated, on his feet. He died on the ninth day. The bladder in this case was ruptured almost in the same situation as in the preceding case, and to the same extent.

*An Account of a Case of Acute Rheumatic Inflammation, terminating in Peritonitis.* By E. M'DOWEL, Licentiate of the Royal College of Surgeons in Ireland.

This case is interesting as an instance of metastasis of disease from one part to another of similar structure. The patient, a woman aged 28, had severe inflammation of the synovial membranes of the knee-joints, which was almost totally relieved by venesection, leeches, &c.; but, after the lapse of a few days, the patient was seized with inflammation of the peritoneum, which, notwithstanding the use of bleeding, &c. rapidly terminated in death. Dissection showed signs of violent inflammation over the whole extent of the peritoneal membrane; there was also much coagulated lymph on the inner surfaces of the synovial membranes of the knee-joints.

Mr. M'Dowel calls the disease rheumatism, and on this foundation adduces objections to the opinions generally admitted on the nature and seat of that affection; but we shall not uselessly occupy our pages by remarks on these ill-founded speculations. It should however be mentioned, that the remedial measures employed appear to have been appropriate; except that, in a pharmaceutical point of view, it was wrong to mingle tartarized antimony with decoction of cinchona; and we cannot discern what were the intentions in giving such a combination of the materia medica.

*A Case of Sudden Death, occasioned by Oxalic Acid.* By JOHN MOLLAN, M.D. one of the Physicians to the Dublin General Dispensary.

The patient, a woman about 45 years of age, took, from accident, a solution of nearly two ounces of oxalic acid: almost immediately afterwards she was attacked with vomiting, and complained of a burning heat in her stomach; she had an evacuation by stool, and passed urine; her face and extremities soon became pale and cold, and the skin was partially bedewed with a cold sweat; she vomited almost incessantly; and expired in about twenty minutes.

The patient had experienced an injury on the right side of the chest, a few days previously. Besides signs of inflammation, the following appearances were witnessed, which may perhaps be connected with the agency of the poison:

"The right auricle and ventricle of the heart were considerably distended: on puncturing the auricle a quantity of air escaped, and the heart immediately collapsed. The blood contained in these cavities was dark and fluid, and had a number of air-bubbles floating on its surface. The parietes of the ventricle were thinner than common, as if they had undergone considerable distension. The left ventricle was less contracted than usual, but in every other respect was perfectly natural.

"The stomach, when drawn out, presented the appearance of incipient putrefaction; at the great bulging extremity, the mere laying hold of it between the fingers was sufficient to separate the serous from the other coats. In its cavity about eight ounces of a dark-brown fluid were contained. The mucous membrane was thickened throughout its entire extent, and presented a mottled appearance, the greater part of a dark-brown or blackish colour, as if blood was effused into its structure; whilst the interstices, particularly along the greater curvature, were similar to a finely-injected membrane. The appearances of disorganization were most remarkable at the bulging extremity; yet I have seen the mucous coat separate with more facility in other morbid states of the stomach. The mucous coat of the duodenum, as far as its second curvature, presented an appearance similar to that of the stomach, but less strongly marked. The jejunum, and other intestines, were of the natural appearance. The liver and spleen presented nothing particular. We were not permitted to examine the head."

*Cases of Fracture of the Neck of the Femur, illustrated by Dissections.* By A. COLLES, M.D. one of the Professors of Anatomy and Surgery in the Royal College of Surgeons in Ireland, &c. &c.

We shall transcribe some of the cases, which comprise the more general observations made by Dr. Colles on this subject.

"*No.* I.—This was an old man, whose body was brought to the theatre of the College of Surgeons for dissection. We were totally unacquainted with the history of the fracture, which was found in the left femur.

"The capsular ligament was remarkably increased in thickness and in closeness of texture. The fracture had taken place in the neck of the bone, near to the trochanter, but still within the capsular ligament.

"The fractured surface of the upper piece exhibited many spots apparently covered with a cartilaginous incrustation: these appearances, on a more close examination, were found to be owing to the conversion of small portions of the bone into a substance resembling ivory. The lower fractured surface, widely expanded, was formed into a sort of cup, as if the bone had been rendered soft, and, while in that state, had been acted upon by the upper piece, which was pressed on it by the weight of the body. One part of the edge of this cup-like surface was formed of two pretty large fragments of bone, which were closely connected to it by a strong ligamentous substance.

"The round head of the bone was retained in the acetabulum by the ligamentum teres, which remained entire. The cartilaginous incrustation of its head was deficient in two or three small patches, and here the bony structure was uncovered. On the edge of one of these spots was a raised part, as if a small fragment had formerly been broken off, and had adhered to the bone, without returning perfectly to its former level.

"No intermediate substance held the fractured surfaces in apposition, each being connected with the capsular ligament by very strong ligamentous bands, which passed from the internal surface of the capsule to almost every point of the outer surface of the fractured pieces. Hence it is obvious that no effort of nature had been made to create a re-union between the two pieces of the fracture, and that the stability of the limb had depended upon the strength of those ligamentous bands by which each piece was connected with the capsular ligament of the joint, aided, no doubt, by the extraordinary thickness which the capsule had acquired.

"*No.* III.—The following appearances were observed in the left thigh of a female subject, brought into the dissecting-room of the College of Surgeons, March 1818.

"The left thigh-bone was fractured transversely, and on a level with the brim of the acetabulum. Two strong ligamentous bands, one arising from the edge of the acetabulum, and the other from the internal surface of the capsular ligament, stretched across to the broken surface of the head of the bone, and seemed as if they had assisted the round ligament in confining the head in the socket. The head of the bone was perfectly sound, as was the ligamentum teres.

"The two surfaces of the fracture anteriorly admitted of a separation from each other to the extent of an inch, having at this part no other connexion than two or three tendinous bands, nearly an inch long, and very distant from each other. Posteriorly, these surfaces were united together by a very strong ligamentous substance, which was so connected with the capsular ligament, that it appeared as if it were formed by the ligament sending a thick production across between the fractured surfaces. At this place the capsular ligament did not morbidly adhere to the neck of the thigh-bone.

"The neck of the femur was evidently shortened, the lower surface of the fracture appearing expanded, as if it had yielded to the pressure of great weight.

"The broken surface of the head had occasionally moved on the shaft of the femur, as low down as the small trochanter.

"*No.* VII.—The fracture was transverse, and close to the head of the femur. The capsular ligament was very much thickened, and its internal surface coated with coagulated lymph. The fracture, however, was incomplete; for the external bony coating of the neck of the femur remained unbroken for nearly half the circumference of the bone at its posterior part, and was reduced to the softness and whiteness of cartilage. To the internal surface of this unbroken portion adhered many bony fragments of different sizes, which, by the violence of the fracture, appeared to have been torn away from the reticular substance of the bone, retaining their connexion with this coating. Along the anterior part of the fracture, where the external coating had been broken, we found its torn edges projecting beyond the fractured surface, in some places to a height of a quarter of an inch, in others less, and all those projecting portions reduced to the softness of a membrane.

"At that part of the head of the femur, which in the ordinary position would correspond with the posterior origin of the rectus femoris, a portion of bone appeared to be wanting. Whether this had been caused by a splintering of bone, or had been produced by some process which took place after the fracture, is uncertain; but I am disposed to ascribe it to the former, for still higher up on the bone was an appearance of a fissure. All this part, from which it may be supposed the splinter was detached, was covered by a thick and vascular membrane, extending from the fissure down to the edge of the fracture, so that little more than the mere edge presented a bare surface; the capsular ligament corresponding with this surface was very much thickened. Near the centre of the fractured surface of the upper piece, a small portion of very solid bone appeared, as if it had been forcibly driven into the cancellated structure of this part of the bone. The round ligament was highly vascular and inflamed. Scarcely any vestige of a membranous texture could be discovered on the broken surface of the upper piece; the corresponding surface of the lower piece was only in some places covered with a membrane.

"*No.* X.—This bone was found in an adult subject, whose body was brought into the dissecting-room.

"A vertical section of the bone presented the following appearances: The head perfectly healthy and natural; the angle formed by the neck and shaft of the bone differed very little from the natural angle. The neck, of full length, was laid across the extremity of the shaft in such a manner, that the fractured end of the upper solid plate of the neck lay in contact with the end of the external plate of the shaft; while the lower solid wall of the neck passed to almost midway within the canal of the shaft: this lower wall had lost considerably of its compact texture, as if resolving itself into large cancelli. The medullary canal at this place was crossed by a partition of solid bone, on which the extremity of the neck rested. Immediately below this transverse bony partition, the medullary canal appeared destitute of cancellated structure, and extremely vascular, as far as its cavity was exposed.

"A thin blue layer of a substance intermediate between ligament and cartilage, was everywhere interposed between the neck and shaft of the bone, and also between the neck and the new irregular bony masses. A similar bluish cartilaginous substance was also interposed between the different portions of the irregular masses.

"The neck of the bone retained its full length and size. After maceration, it appeared that the periosteum of the neck remained unaltered.

Amongst the remarks and conclusions of Dr. Colles on this subject, he observes,

"The circumstances which I found common to all these fractures were, that the capsular ligament was not lacerated, (except, perhaps, in No. 5.) Hence may be inferred the fallacy of Sabatier's reasoning, in ascribing the pain which is occasioned by moving the fractured limb to the friction of the broken surface against the flesh *(chairs)* in its vicinity. In every instance I remarked the increased thickness of the capsule. It is curious that, although this membrane exhibited in eleven cases such manifest proofs of previous inflammation, yet in none of them could we discover any adhesion between it and the outer surface of the neck of the bone."

We would inform such of our readers as may not be well acquainted with the idiom of the French language, that the word *chairs* (plural) is used precisely in the same sense as we employ the expression "*the soft parts;*" and therefore the critical remark of Dr. Colles on the opinions of Professor SABATIER is not well-founded.

*Observations on the Operation for Artificial Pupil, illustrated by Cases and Engravings.* By E. RYAN, M.D. Senior Surgeon to the Kilkenny County Hospital, &c.

The remarks contained in this paper are deduced from extensive experience and apparent accuracy of observation, and appear to be judicious and well-founded; but, as the subject is not generally interesting, we shall not enter into a particular consideration of them.

On closing this volume, we observe, that, being principally composed of histories of facts, but little interspersed with arguments, the remarks of the critic must be nearly confined to such as relate to the estimation of the value of those facts: the transcriptions we have given from it will show that we recognise in it much very valuable matter; but the proportion of common-place observations which it also contains, shows that a more severe selection on the part of the editors will be necessary to preserve to the work the high character it has justly acquired.

---

*Cases of Hydrophobia.* By GEORGE PINCKARD, M.D. Deputy Inspector-General of Hospitals to his Majesty's Forces; Physician to the Bloomsbury Dispensary, the London Female Penitentiary, &c. &c. 8vo. pp. 38. Callow, 1819.

We shall introduce this work to our readers by the words of the author in the preface: he says,

"Amidst the conflicting opinions of the medical world, respecting the nature and treatment of a disease so ill-understood, so uniformly fatal, and (comparatively) so seldom witnessed, as hydrophobia, it were to be wished that physicians felt it a duty to record every case which presented itself to their observation.

"Under this impression I noted minutely the occurrences in a case which was lately brought under my care at Battle-bridge, intending to give them to the public through the medium of some periodical work. But it has been suggested to me, that, as three other cases* of this untractable disease have fallen under my observation at different periods, it might be more satisfactory to collect them together, and publish them all under the same cover.

"Adopting this suggestion, I shall content myself with plainly narrating the history of the several cases, leaving the reader to draw such conclusions as the circumstances may seem to warrant.

"At present I am inclined to believe, that all the curative means which have been tried are equally inefficacious: still, by publishing the cases which occur, facts may be developed which may lead to a better knowledge of the disorder; and it is reasonable to hope, that,

* These were published at different times in the *London Medical Journal.*

whatever contributes towards establishing the true character of the disease, brings us nearer to the discovery of a remedy.

"One fact of high importance may be remarked in the case of Mr. Hubbard: that the disease supervened, notwithstanding the free application of caustic to the wound, within less than twenty-four hours after it was inflicted; whence it appears that each moment of delay is fraught with extreme hazard; and that in every instance the sovereign preventive, a complete excision of the part, or the most perfect destruction by caustic† or the actual cautery, should be effected as speedily as possible."

These sentiments will be echoed by every true physician; and the zeal of the author to promote the elucidation of the malady to which they relate, must be generally applauded. We have not, it is true, hitherto acquired any precise etiological knowledge of it, but we must not therefore suffer our hopes to be depressed: that of tetanus was, until a few years since, as little known; but, by pursuing the course of which Dr. Pinckard has here given so good an example, that of tracing, accurately, a description of the morbid phenomena during life, and the physical appearances after death, in all cases, whether or not any thing novel or peculiar might be obvious in them, the nature of the latter disease has at length been ascertained.

We regret to find that some cases of hydrophobia have recently occurred, of which histories have not been published by the medical attendants; and still more so, that examination of the dead bodies has yet oftener not been made. Permission to effect this should be urged in every instance, and we would on this point offer one suggestion,—that the centres of the ganglionic system of nerves, especially the cœliac plexus, be accurately examined. Reasoning by induction from the symptoms, we are led to suspect that a morbid state of this part of the nervous system is a chief, if not an essential, cause in the production of the phenomena witnessed in this disease; and no notice is taken of the state of it in any of the cases on record.

The histories of Dr. Pinckard must be considered of much value, being related with perspicuity, and deduced from comprehensive views. If we ever discover the primary morbid lesion, the advantage of possessing such histories as these will then be more clearly apparent: we shall probably find every remark in them illustrative of the nature and progressive development of the train of phenomena evinced in this malady.

---

† To insure an effectual application of the caustic to every part of the wound, might it not be advisable to use it in a liquid form, such as the muriatic, nitric, or sulphuric, acid?

